# Hematological Inflammatory Markers in Patients with Clinically Confirmed Familial Hypercholesterolemia

**DOI:** 10.1155/2022/5051434

**Published:** 2022-01-17

**Authors:** Golnaz Vaseghi, Kiyan Heshmat-Ghahdarijani, Marzieh Taheri, Ghazal Ghasempoor, Shabnam Hajian, Shaghayegh Haghjooy-Javanmard, Roqayeh Aliyari, Zahra Shafiee, Masoud Shekarchizadeh, Ali Pourmoghadas, Nizal Sarrafzadegan

**Affiliations:** ^1^Applied Physiology Research Center, Cardiovascular Research Institute, Isfahan University of Medical Sciences, Isfahan, Iran; ^2^Heart Failure Research Center, Cardiovascular Research Institute, Isfahan University of Medical Sciences, Isfahan, Iran; ^3^Cardiovascular Research Institute, Isfahan University of Medical Sciences, Isfahan, Iran; ^4^Department of Biostatistics, School of Medical Sciences, Tarbiat Modares University, Tehran, Iran; ^5^Isfahan Cardiovascular Research Center, Cardiovascular Research Institute, Isfahan University of Medical Sciences, Isfahan, Iran

## Abstract

**Background and Aims:**

Familial hypercholesterolemia (FH) is an autosomal dominant genetic disorder of lipid metabolism which leads to premature cardiovascular diseases. In patients with FH, blood inflammatory markers may be disrupted; however, its extent is unclear. In this study, we aimed to evaluate the NLR (neutrophil to lymphocyte ratio), PLR (platelet count to lymphocyte count ratio), MPV (mean platelet volume), RPR (red blood cell distribution width to platelet count ratio), WBC (white blood cell), and PDW (platelet distribution width and platelet count).

**Methods:**

The patients were selected from laboratories due to high cholesterol level and who had history of premature cardiovascular disease. The Dutch Lipid Clinic Network (DLCN) criteria are used for the detection of FH. Controls had a history of hyperlipidemia, and both groups could be on pharmacotherapy or not. All the biochemical markers were evaluated using appreciate methods. Statistical analysis was done using STATA 14.

**Results:**

The study group consisted of 1074 patients with FH and 473 control cases. Of the CBC inflammatory markers, only PLR was significantly (*p* value = 0.003) higher in FH patients (7.96 ± 10.08) compared to non-FH (6.45 ± 2.44). In FH patients, the PLR was significantly higher in probable/definite FH group (9.70 ± 14.06) compared to possible FH (7.36 ± 8.23) (*p* value < 0.001). Linear regression analysis showed that only RLR was independently associated with total cholesterol (*b* = 0.000, *p* = 0.13).

**Conclusions:**

Our results may show the importance of high cholesterol on platelet activity and highlight the use of lipid lowering drugs in patients with hyperlipidemia.

## 1. Introduction

Familial hypercholesterolemia (FH) is a monogenic disorder which inherited in an autosomal dominant trait [1]. Lipoprotein metabolism is impaired in FH and results in severe elevation of low-density lipoprotein cholesterol (LDL-C) concentration. Patients with FH have the greatest risk of premature cardiovascular disease (CVD) [1]. A mutation in LDL receptor gene, Apo lipoprotein (Apo) B100 gene, or proportion convertase subtilisin/kexin type 9 (PCSK9) can be identified in 30% to 80% of patients with clinically diagnosed FH. Alternatively, about 20% of clinical FH is thought to have a polygenic cause [2].

Molecular diagnosis is recommended for FH patients but still not easily available, and also, recent reclassification of genetic variants associated with FH limits its routine use. Therefore, FH remains subdiagnosed and inadequately treated till now. New FH clinical diagnostic criteria like FAMCAT are being tested and seem to be more accurate than the classical ones [3]. It has been shown that FH is associated with inflammation, endothelial activation, and oxidative stress [4, 5].

The complete blood cell (CBC) count, an easily available test, is used wildly in clinical practice. Recently, the indices derived from CBC are recognized as novel inflammatory markers and predictors of outcome in chronic inflammatory diseases [6] and risk predictor for coronary heart disease [7]. Mean platelet volume (MPV), platelet distribution width (PDW), and platelet-to-lymphocyte ratio (PLR) are new inflammatory markers which have been recognized for the assessment of inflammation and endothelial dysfunction in many inflammatory and cardiovascular diseases [8]. The neutrophil–lymphocyte ratio (NLR), another CBC derived marker, is considered to evaluate systemic inflammation and endothelial function. It has been shown that NLR is associated with the severity of coronary artery disease [9].

Animal studies show the link between dyslipidemia and increased leukocytosis; however, this association in human has not been well understood [10]. It has been shown that increased triglyceride is associated with increased total white blood cell, lymphocyte, neutrophil, and monocyte counts [11]. It also has been reported that increased LDL cholesterol levels are related to increased lymphocyte numbers [12, 13] but lower total white blood cell and monocyte and neutrophil counts [14]. A positive correlation between non-HDL cholesterol levels and platelet counts has also been reported [15].

Because FH patients are at greater risk of developing atherosclerosis-related diseases and in earlier aged, the need for early diagnosis is really important to initiate appropriate aggressive treatment. Therefore, we aimed to investigate some hematologic inflammatory factors in patients with FH to find any association for early diagnosis that might need actions.

## 2. Method

### 2.1. Study Population

The patients in this study were selected from IRFH (Isfahan registry of FH) [16]. Briefly, the enrollment framework in these approaches was based on first investigating laboratories for contacting patients with high LDL-C to enroll them in our study (National Clinical Trial No. 2865694). All individuals aged above 2 years irrespective of their sex with LDL-C of more than 150 mg/dl (LDL − C > 190 mg/dl or LDL − C > 150 mg/dl but under pharmacological treatment) were contacted by phone to come to our clinic for further evaluation. We used the Dutch Lipid Clinic Network Score (DLCNS), which was based on the clinical symptoms of FH and family history. Patients who were clinically diagnosed with definite or probable FH were enrolled in the study according to the DLCNS as previously described [17].

Key exclusion criteria were all causes of secondary hyperlipidemia such as hypothyroidism, liver and kidney disease, and medicine which affect lipid profile. All the eligible individuals signed the constant form. The control group was selected from the patients with a history of hyperlipidemia but not diagnosis with FH. Patients on pharmacotherapy were not excluded from our study. Hypertension was defined as blood pressure ≥ 140/90 mmHg, and diabetes mellitus was defined as two fasting glucose levels > 126 mg/dl [18].

### 2.2. Biochemical Measurement

Venous blood samples were taken from patients after ≥8 hours of overnight fasting. High-density lipoprotein (HDL-C) and serum total cholesterol and triglycerides (TG) were measured by enzymatic assays (Boehringer, Mannheim, Germany). LDL-C was measured by the LDL Cholesterol Assay Kit (Birex-Fars). The automated machine (Hitachi 902) was used to perform fasting blood sugar (FBS) and white blood cell count (WBC). Other factors were measured with standard methods. All measurements were performed at the hospital's central laboratory.

### 2.3. Statistical Analysis

Continuous and categorical data are reported as means ± SD and frequency (percentage), respectively. Demographical, clinical, and biochemical characteristics compared between FH and non-FH participants using independent sample *t*-test or one-way analysis of variance and chi-square test, for continuous and categorical variables, respectively. For comparing inflammatory and biochemical markers between FH and non-FH participants and also to determine the relationship between cholesterol, LDL-C, and inflammatory markers, since participants may be related to each other (family members), linear random intercept model was used to consider dependencies and eliminate effects. Also, multiple linear random intercept models were used to compare inflammatory markers between FH and non-FH groups adjusting hierarchically, first for age, BMI, and smoking status and then additionally for antilipid drug, aspirin consumption, history of CVD, history of diabetes, and finally total cholesterol. For all models, *β* (95% CI) was reported. Statistical analysis was done using STATA 14 (Stata Corp, College Station, Texas, USA). *p* value < 0.05 considered statistically significant.

## 3. Result

The demographic and clinical characteristics of patients with FH and non-FH group are summarized in Table 1. The study population included 473 control cases and 1074 FH patients (0.78% possible FH and 22% definite/probable according to Dutch Lipid Clinic definition). The mean of age of all participants was 47.5 ± 13.3, and in FH group (total), possible FH, probable/definite FH, and non-FH groups were 45.5 ± 14.8, 50.3 ± 11.7, 50.3 ± 13.9, and 50.3 ± 12.3, respectively. All participants were between 2 and 75 years old. There were 59.4% male in non-FH group and 53.4% in FH group. As it is shown in Table 1, there were no statistically significant differences between the two groups in terms of basic characteristics including gender, smoking, antiplatelet drug use, DM type 1, and the level of TG and HDL; however, these two groups showed significant differences in terms of age, BMI, serum LDL, total cholesterol, FBS, LDL/HDL ratio, DM type 2, history of CVD, hypertension, and use of lipid-lowering therapy (LLT).

Table 2 shows the CBC derived inflammatory markers in FH and non-FH patients in our study. There was a significant difference between the two groups only in terms of PLR as it was significantly higher in FH group (7.96 ± 10.08) compared to non-FH (6.45 ± 2.44) group (*p* value = 0.003). In FH patients, the PLR was significantly higher in probable/definite FH group (9.70 ± 14.06) compared to possible FH (7.36 ± 8.23) (*p* value < 0.001). The NLR, WBC, PDW, and platelet count were higher in FH group compared with control but not significantly (*p* value = 0.196, *p* value = 0.196, and *p* value = 0.086, respectively). RPR was lower in FH group (0.02 ± 0.01) than non-FH (0.02 ± 0.02) but the difference was not significant (*p* value = 0.212).

As shown in Table 3, the correlation between hematological inflammation factors, i.e., PLR, PRP, NLR, WBC and PDW, was adjusted in 4 different models: model 1 which was crude effect; model 2 which was adjusted for age, BMI, and smoking statues; model 3 which was adjusted for antilipid drug, aspirin consumption, history of CVD, and history of diabetes; and the model 4 which was adjusted for cholesterol. In all these four models, PLR was meaningfully different between FH and non-FH groups and also between possible and definite and probable groups (*p* value < 0.05).

Our analysis using linear regression showed that only RLR was independently associated with total cholesterol in FH group (0.003(0.001,0.005)) (*p* value < 0.001) and possible FH group (0.004(0.002,0.007)) (*p* value < 0.001) but not in non-FH group (0.001(-0.00,0.001)) (*p* value = 0.078) after multi-multiple adjustment. (Table 4).

## 4. Discussion

This study evaluated the colorations of CBC inflammatory markers in FH patients for the first time. In the current study from FH population, after multiple adjusting for relevant covariates, we provided evidence that there was a significant coloration between platelet-to-lymphocyte ratio (PLR) and cholesterol in FH patients. Our results showed no significant differences between the two groups in terms of other CBC derived inflammatory markers. In this study, patients with different levels of LDL-C were studied, so that some of them were FH patients with decreased LDL-C level and some of them were patients with high and irregular hyperlipidemia who were not FH. High levels of serum cholesterol and LDL-C lead to premature atherosclerosis at early age and also increase the production of cellular adhesion molecules and proinflammatory cytokines [19]. FH patients also have endothelial dysfunction [20], which can be explained with inflammatory nature of disease.

NLR, PLR, and RPR are known as the hematological markers of systemic inflammation and also as positive predictors of CVDs. These three might play a role as repetitive and simple markers of peripheral artery disease. NLR is used to determine the severity of inflammation [21]. Till now, it has been shown that some diseases such as diabetes mellitus, thyroid functional abnormalities, and some malignancies may affect the NLR, but in the present study, NLR was not significantly different between patients with and without FH. RPR is another valuable laboratory test used to predict the mortality in some diseases such as hepatic fibrosis and cirrhosis [22, 23]. This factor also did not show a significant relationship with any of the variables in our study. Our results after multiple adjustment showed that only PLR is associated with higher cholesterol in FH patients. PLR was found to be an independent prognostic risk factor in patients with malignancies such as pancreatic or colorectal cancer [24]. Increased level of PLR was showed to be significantly linked with higher risk of critical limb ischemia (CLI) and other cardiovascular endpoints. Significant association between decreased HDL-C level and increased PLR level has been reported by Prajapati et al. in those with angiographically confirmed coronary blockages and also in healthy, young individuals [25]. Platelets are critical components of inflammation, atherothrombosis, and atherosclerosis which therefore play an important role in vascular health. Icli et al. showed that MPV has been increased in patients with FH and is associated independently with total cholesterol level [26]. Jagroop et al. showed that platelet cholesterol (PC) could be correlated with serum LDL-C and total cholesterol. They found that an increased in PC content may affect platelet membrane fluidity, thereby resulting in platelet hyperactivity [27]; however, our results showed no meaningful differences between MPV in FH and control groups. This discrepancy between our results and the previous study may be due to differences in the control groups because in the previous study the control group was subjects with norm lipid levels, but in our study, the control was non-FH dislipidemic patients; however, in both studies, the platelet count was different between the groups.

Genetic examination is still considered as the gold test for FH diagnosis; however, it is not always possible to perform this test due to high cost of it. So far, except genetic test, the Dutch Lipid Clinic Network (DLCN) is the most widely accepted criteria in terms of clinical manifestation. Our study showed that this simplified screening algorithm has been successful in terms of identifying FH patients and discriminating them form non-FH dyslipidemic patients. However, using blood test is still in its infancy, and these tests do not have specificity for FH detection; other blood test should be added and determine its accuracy for FH detection.

It has been suggested that higher total cholesterol is associated with lower total white blood cell count and also lower monocyte and neutrophil count. The regression analysis proposed that both associations may be more important at lower total cholesterol levels and flatter at higher total cholesterol levels, with a threshold at approximately 155 and 204 mg/ld., respectively [14]. In our study, all patients had history of hypercholesterolemia, and we did not observed differences between their WBC count; however, we observed significant differences when compare inflammation markers consists of WBC component and platelet count based on LDL-C and cholesterol level.

### 4.1. Limitations of the Study

First, study data were collected from one center and also limited population. Second, we did not conduct a genetic test to confirm FH. Third, we included the patients who received any lipid-lowering agent which may affect the sample size after adjustment.

## 5. Conclusion

In conclusion, our findings show that PLR is significantly associated with higher cholesterol in patients with FH, which emerge the treatment of hyperlipidemia for any reason.

## Figures and Tables

**Table 1:** Demographical, clinical, and biochemical characteristics of FH and non-FH patients.

Parameter	FH group			*p* value	FH group		*p* value
	Non-FH (473)	Possible (813)	Definite and probable (261)		FH (1074)	Non-FH	
Age^∗∗^	45.5 ± 14.8	50.3 ± 11.7	50.3 ± 13.9	<0.001	45.5 ± 14.8	50.3 ± 12.3	<0.001
Sex^∗^ (male)	281 (59.4)	452 (55.7)	110 (42.1)	<0.001	562 (52.4)	281 (59.4)	0.011
Smoking^∗^ (smokers)	49 (10.4)	133 (16.4)	29 (11.1)	0.004	162 (15.1)	49 (10.4)	0.013
HTN^∗^	144 (30.5)	348 (43.0)	88 (34.5)	<0.001	436 (40.9)	144 (30.5)	<0.001
Type2-DM^∗^	57 (12.1)	170 (21.1)	58 (23.3)	<0.001	228 (21.6)	57 (12.1)	<0.001
CVD history^∗^	139 (29.4)	432 (53.2)	124 (47.5)	<0.001	556(51.8)	139 (29.4)	<0.001
Aspirin use^∗^	117 (39.9)	284 (77.2)	63 (67.0)	<0.001	347 (75.1)	117 (39.9)	<0.001
Antilipid drug^∗^	178 (37.9)	495 (64.0)	180 (77.3)	<0.001	675 (67.0)	178 (37.9)	<0.001
BMI (kg/m^2^)^∗∗^	27.0 ± 5.9	28.1 ± 10.2	27.6 ± 5.2	0.015	28.0 ± 9.3	27.0 ± 5.9	0.002
Total cholesterol (mg/dl)^∗∗∗^	178.6 ± 43.7	198.9 ± 59.2	244.5 ± 90.1	<0.001	210.0 ± 70.8	178.6 ± 43.7	<0.001
LDL (mg/dl)^∗∗∗^	99.9 ± 30.3	115.4 ± 43.8	149.3 ± 68.6	<0.001	123.7 ± 52.9	99.9 ± 30.3	<0.001
Triglyceride (mg/dl)^∗∗∗^	150.1 ± 79.8	157.2 ± 68.7	163.1 ± 75.2	0.009	158.6 ± 70.3	150.1 ± 79.8	0.032
HDL (mg/dl)^∗∗∗^	46.4 ± 11.6	46.9 ± 11.5	48.1 ± 12.1	0.021	47.2 ± 11.7	46.4 ± 11.6	0.086
HDL/LDL ratio^∗∗∗^	2.3 ± 0.8	2.6 ± 1.1	3.2 ± 1.3	<0.001	2.7 ± 1.1	2.3 ± 0.8	<0.001

^∗^Data are shown as frequency (percentage). Chi-square test was used. ^∗∗^Data are shown as mean ± SD. Independent sample *t*-test or one-way analysis of variance was used. ^∗∗∗^Linear random intercept model was used.

**Table 2:** Inflammatory markers of FH and non-FH patients.

Parameter	FH group			*p* value	FH group		*p* value
	Non-FH (473)	Possible (813)	Definite and probable (261)		FH (1074)	Non-FH	
NLr^∗^	1.50 ± 0.76	1.57 ± 1.26	1.58 ± 0.99	0.195	1.57 ± 1.20	1.50 ± 0.76	0.196
PLr^∗^	6.45 ± 2.44	7.36 ± 8.23	9.70 ± 14.06	<0.001	7.96 ± 10.08	6.45 ± 2.44	0.003
RPr^∗^	0.02 ± 0.02	0.02 ± 0.01	0.02 ± 0.02	0.302	0.02 ± 0.01	0.02 ± 0.02	0.212
WBC^∗^10^3^^∗^	6.48 ± 5.17	6.25 ± 2.02	6.17 ± 3.29	0.216	6.23 ± 2.40	6.48 ± 5.17	0.196
PDW^∗^	11.13 ± 1.82	11.20 ± 2.06	11.23 ± 1.84	0.263	11.28 ± 1.84	11.13 ± 1.82	0.085

^∗^Data are shown as mean ± SD. Linear random intercept model was used.

**Table 3:** Comparison of crude and adjusted inflammatory markers between FH and non-FH participants.

Parameter		Comparing non-FH, possible, and definite and probable group		Comparing FH and non-FH	
		*β* (95% CI)	*p* value	*β* (95% CI)	*p* value
NLr	Model 1	0.05 (-0.025, 0.125)	0.195	0.07 (-0.04, 0.18)	0.196
	Model 2	0.05 (-0.034, 0.128)	0.253	0.06 (-0.06, 0.17)	0.337
	Model 3	-0.02 (-0.091, 0.052)	0.596	-0.05 (-0.15, 0.05)	0.377
	Model 4	-0.01 (-0.09, 0.08)	0.902	-0.03 (-0.14, 0.08)	0.551

PLr	Model 1	0.21 (0.105, 0.311)	<0.001	0.23 (0.08, 0.38)	0.003
	Model 2	0.18 (0.069, 0.299)	0.002	0.19 (0.02, 0.36)	0.026
	Model 3	0.07 (0.018, 0.118)	0.007	0.08 (0.01, 0.15)	0.032
	Model 4	0.06 (0.01, 0.12)	0.029	0.07 (-0.01, 0.14)	0.013

RPr	Model 1	-0.001 (-0.002, 0.001)	0.302	-0.001 (-0.003, 0.001)	0.212
	Model 2	-0.001 (-0.002, 0.0001)	0.096	-0.002 (-0.004, 0.000)	0.052
	Model 3	0.001 (-0.001, 0.002)	0.478	0.0001 (-0.002, 0.002)	0.948
	Model 4	0.002 (-0.0002, 0.004)	0.079	0.001 (-0.001, 0.004)	0.431

WBC^∗^10^3^	Model 1	-0.17 (-0.44, 0.10)	0.216	-0.26 (-0.67, 0.14)	0.196
	Model 2	-0.14 (-0.44, 0.16)	0.353	-0.26 (-0.70, 0.18)	0.252
	Model 3	-0.34 (-0.93, 0.25)	0.259	-0.45 (-1.29, 0.39)	0.291
	Model 4	-0.27 (-0.95, 0.41)	0.431	-0.36 (-1.26, 0.54)	0.437

PDW	Model 1	0.08 (-0.06, 0.23)	0.263	0.18 (-0.03, 0.39)	0.085
	Model 2	0.05 (-0.09, 0.20)	0.492	0.12 (-0.10, 0.34)	0.291
	Model 3	0.01 (-0.23, 0.25)	0.940	0.04 (-0.29, 0.38)	0.808
	Model 4	0.01 (-0.27, 0.28)	0.962	0.05 (-0.32, 0.41)	0.807

Model 1: crude effect; Model 2: adjust for age, BMI, and smoking statues; Model 3: additionally, adjusted for antilipid drug, aspirin consumption, history of CVD, and history of diabetes; Model 4: additionally, adjusted for total cholesterol. Data are shown as *β* (95% CI). Linear random intercept model was used.

**Table 4:** Relationship of cholesterol, LDL-C, and inflammatory markers.

Group			PLR		NLR		RPR	
			*β* (95% CI)	*p* value	*β* (95% CI)	*p* value	*β* (95% CI)	*p* value
Cholesterol	FH	Possible	0.004 (0.002, 0.007)	<0.001	0.000 (-0.001, 0.002)	0.606	-0.00002 (-0.00003, -0.00001)	0.001
		Definite and probable	0.001 (-0.003, 0.004)	0.765	-0.00 (-0.002, 0.001)	0.768	-0.00001 (-0.00003, 0.00002)	0.734
		Total	0.003 (0.001, 0.005)	<0.001	0.0002 (-0.001, 0.001)	0.760	-0.00001 (-0.00002, -0.000001)	0.029
	Non-FH		0.001 (-0.00, 0.001)	0.078	-0.003 (-0.004, -0.001)	0.001	-0.00001 (-0.00004, 0.00003)	0.731
	Total		0.003 (0.002, 0.005)	<0.001	-0.0001 (-0.001, 0.001)	0.735	-0.00001 (-0.00002, -0.000001)	0.028

LDL-C	FH	Possible	-0.00003 (-0.00004, -0.00001)	0.001	0.0005 (-0.001, 0.003)	0.609	0.005 (0.002, 0.008)	0.002
		Definite and probable	-0.00001 (-0.00004, 0.00003)	0.668	-0.0003 (-0.002, 0.001)	0.736	0.0007 (-0.004, 0.006)	0.787
		Total	0.0038 (0.0014, 0.0063)	0.002	0.0002 (-0.001, 0.002)	0.790	-0.00001 (-0.00003, -0.000001)	0.035
	Non-FH		0.0006 (-0.0004, 0.0017)	0.244	-0.004 (-0.007, -0.002)	0.001	-0.000004 (-0.00005, 0.00006)	0.897
	Total		0.004 (0.002, 0.006)	<0.001	-0.0002 (-0.001, 0.001)	0.743	-0.00002 (-0.00003, 0.00)	0.050

Data are shown as *β* (95% CI). Linear random intercept model was used.

## Data Availability

Data available on request.

## References

[1] Repas R. B., Tanner J. R. (2014). Preventing early cardiovascular death in patients with familial hypercholesterolemia. *The Journal of the American Osteopathic Association*.

[2] Trinder M., Li X., DeCastro M. L. (2019). Risk of premature atherosclerotic disease in patients with monogenic versus polygenic familial hypercholesterolemia. *Journal of the American College of Cardiology*.

[3] Santos R. D. (2019). Screening and management of familial hypercholesterolemia. *Current Opinion in Cardiology*.

[4] Rahman T., Hamzan N. S., Mokhsin A. (2017). Enhanced status of inflammation and endothelial activation in subjects with familial hypercholesterolemia and their related unaffected family members: a case control study. *Lipids in Health and Disease*.

[5] Upadhyay R. K. (2015). Emerging risk biomarkers in cardiovascular diseases and disorders. *Journal Of Lipids*.

[6] Bilgin S., Aktas G., Zahid Kocak M. (2019). Association between novel inflammatory markers derived from hemogram indices and metabolic parameters in type 2 diabetic men. *The Aging Male*.

[7] Madjid M., Fatemi O. (2013). Components of the complete blood count as risk predictor for coronary heart disease: a review. *Texas Heart Institute Journal*.

[8] Leader A., Pereg D., Lishner M. (2012). Are platelet volume indices of clinical use? A multidisciplinary review. *Annals Of Medicine*.

[9] Arbel Y., Finkelstein A., Halkin A. (2012). Neutrophil/lymphocyte ratio is related to the severity of coronary artery disease and clinical outcome in patients undergoing angiography. *Atherosclerosis*.

[10] Murphy A. J., Akhtari M., Tolani S. (2011). Apoe regulates hematopoietic stem cell proliferation, monocytosis, and monocyte accumulation in atherosclerotic lesions in mice. *The Journal of Clinical Investigation*.

[11] Bernelot Moens S. J., Verweij S. L., Schnitzler J. G. (2017). Remnant cholesterol elicits arterial wall inflammation and a multilevel cellular immune response in humans. *Arteriosclerosis, Thrombosis, and Vascular Biology*.

[12] Oda E. (2014). Longitudinal associations between lymphocyte count and LDL cholesterol in a health screening population. *Journal of Clinical & Translational Endocrinology*.

[13] Oda E., Kawai R., Aizawa Y. (2012). Lymphocyte count was significantly associated with hyper-LDL cholesterolemia independently of high-sensitivity C-reactive protein in apparently healthy Japanese. *Heart and Vessels*.

[14] Lai Y. C., Woollard K. J., McClelland R. L. (2019). The association of plasma lipids with white blood cell counts: results from the multi-ethnic study of atherosclerosis. *Journal of Clinical Lipidology*.

[15] Fessler M. B., Rose K., Zhang Y., Jaramillo R., Zeldin D. C. (2013). Relationship between serum cholesterol and indices of erythrocytes and platelets in the US population. *Journal of Lipid Research*.

[16] Vaseghi G., Arabi S., Haghjooy-Javanmard S. (2019). CASCADE screening and registry of familial hypercholesterolemia in Iran: rationale and design. *ARYA Atherosclerosis*.

[17] Harada-Shiba M., Arai H., Ishigaki Y. (2018). Guidelines for diagnosis and treatment of familial hypercholesterolemia 2017. *Journal of Atherosclerosis and Thrombosis*.

[18] Alberti K. G., Zimmet P. Z., WHO Consultation (1998). Definition, diagnosis and classification of diabetes mellitus and its complications. Part 1: diagnosis and classification of diabetes mellitus provisional report of a WHO consultation. *Diabetic Medicine*.

[19] Hovingh G. K., Davidson M. H., Kastelein J. J., O’Connor A. M. (2013). Diagnosis and treatment of familial hypercholesterolaemia. *European Heart Journal*.

[20] Stokes K. Y., Cooper D., Tailor A., Granger D. N. (2002). Hypercholesterolemia promotes inflammation and microvascular dysfunction: role of nitric oxide and superoxide^1^. *Free Radical Biology & Medicine*.

[21] Condado J. F., Junpaparp P., Binongo J. N. (2016). Neutrophil-lymphocyte ratio (NLR) and platelet-lymphocyte ratio (PLR) can risk stratify patients in transcatheter aortic-valve replacement (TAVR). *International Journal of Cardiology*.

[22] Shuster L. T., Rhodes D. J., Gostout B. S., Grossardt B. R., Rocca W. A. (2010). Premature menopause or early menopause: long-term health consequences. *Maturitas*.

[23] Chen B., Ye B., Zhang J., Ying L., Chen Y. (2013). RDW to platelet ratio: a novel noninvasive index for predicting hepatic fibrosis and cirrhosis in chronic hepatitis B. *PLoS One*.

[24] Betterle C., Volpato M., Rees Smith B. (1997). I. Adrenal cortex and steroid 21-hydroxylase autoantibodies in adult patients with organ-specific autoimmune diseases: markers of low progression to clinical Addison’s disease. *The Journal of Clinical Endocrinology and Metabolism*.

[25] Tok D., Iscen S., Ozenc S. (2014). Neutrophil—lymphocyte ratio is associated with low high-density lipoprotein cholesterol in healthy young men. *SAGE Open Medicine*.

[26] Icli A., Aksoy F., Nar G. (2016). Increased mean platelet volume in familial hypercholesterolemia. *Angiology*.

[27] Jagroop I. A., Persaud J. W., Mikhailidis D. P. (2011). A new rapid method to measure human platelet cholesterol: a pilot study. *Clinical and Applied Thrombosis/Hemostasis*.

[28] Vaseghi G., Heshmat-Ghahdarijani K., Shafiee Z. (2020). *Hematological inflammatory markers in patients with clinically confirmed familial hypercholesterolemia*.

