# Corrigendum to “Hematological Inflammatory Markers in Patients with Clinically Confirmed Familial Hypercholesterolemia”

**DOI:** 10.1155/2023/9851784

**Published:** 2023-04-05

**Authors:** Golnaz Vaseghi, Kiyan Heshmat-Ghahdarijani, Marzieh Taheri, Ghazal Ghasempoor, Shabnam Hajian, Shaghayegh Haghjooy-Javanmard, Roqayeh Aliyari, Zahra Shafiee, Masoud Shekarchizadeh, Ali Pourmoghadas, Nizal Sarrafzadegan

**Affiliations:** ^1^Applied Physiology Research Center, Cardiovascular Research Institute, Isfahan University of Medical Sciences, Isfahan, Iran; ^2^Heart Failure Research Center, Cardiovascular Research Institute, Isfahan University of Medical Sciences, Isfahan, Iran; ^3^Cardiovascular Research Institute, Isfahan University of Medical Sciences, Isfahan, Iran; ^4^Ophthalmic Epidemiology Research Center, Shahroud University of Medical Sciences, Shahroud, Iran; ^5^Department of Biostatistics, School of Medical Sciences, Tarbiat Modares University, Tehran, Iran; ^6^Isfahan Cardiovascular Research Center, Cardiovascular Research Institute, Isfahan University of Medical Sciences, Isfahan, Iran

In the article titled “Hematological Inflammatory Markers in Patients with Clinically Confirmed Familial Hypercholesterolemia” [1], the author Roqayeh Aliyari was shown to be affiliated solely to the Department of Biostatistics, School of Medical Sciences, Tarbiat Modares University, Tehran, Iran.

However, the author was also affiliated to the Ophthalmic Epidemiology Research Center, Shahroud University of Medical Sciences, Shahroud, Iran.

The corrected list of affiliations is shown in the author information above.
